# Comparative prognostic implication of treatment response assessments in mCRPC: PERCIST 1.0, RECIST 1.1, and PSA response criteria

**DOI:** 10.7150/thno.39838

**Published:** 2020-02-10

**Authors:** Erik M. Velez, Bhushan Desai, Lingyun Ji, David I. Quinn, Patrick M. Colletti, Hossein Jadvar

**Affiliations:** 1 Division of Nuclear Medicine, Department of Radiology, Keck School of Medicine of USC, University of Southern California, Los Angeles, California; 2 Department of Preventive Medicine, Keck School of Medicine of USC, University of Southern California, Los Angeles, California; 3 Division of Oncology, Department of Medicine, Kenneth J. Norris Jr. Comprehensive Cancer Center, Keck School of Medicine of USC, University of Southern California, Los Angeles, California

**Keywords:** ^ 18^F-FDG, PET/CT, Prostate, Cancer, Metastatic, Castrate-resistant

## Abstract

Accurate appraisal of treatment response in metastatic castrate-resistant prostate cancer (mCRPC) is challenging in view of remarkable tumor heterogeneity and the available choices among many established and novel therapeutic approaches. The purpose of this single-center prospective study was to evaluate the comparative prognostic utility of PERCIST 1.0 in predicting overall survival (OS) in patients with mCRPC compared to RECIST 1.1 and prostate-specific antigen (PSA)-based treatment response assessments.

**Methods**: Patients with mCRPC were prospectively enrolled if they were beginning systemic medical therapy or transitioning to new systemic therapy after not responding to a prior treatment. All patients underwent a baseline ^18^F-fluorodeoxyglucose (FDG) positron emission tomography/ computed tomography (PET/CT) prior to the initiation of treatment and again 4 months after the start of therapy. Patients' responses to treatment at 4 months compared to baseline were evaluated with RECIST 1.1, PERCIST 1.0 and PSA response criteria. The associations between patients' response categories and OS were evaluated. OS was defined as the duration in time between the date of baseline PET/CT to death from any cause. Patients with different response status were compared with logrank tests. Survival probabilities were calculated using the Kaplan-Meier method.

**Results**: Patients with progressive disease by PSA response criteria at 4 months demonstrated significantly shorter OS (24-month OS probability: 18% ± 11%) compared to patients with stable disease, SD, (44% ± 19%, p=0.03) and complete response, CR, or partial response, PR, (53% ± 11%, p=0.03). RECIST 1.1 response criteria demonstrated a similar trend in OS, however no statistically significant differences were noted between patients with PD (25% ± 15%) compared to SD/non-CR, non-PD (54% ± 13%) and CR/PR (54% ± 14%) (p=0.13). PERCIST 1.0 criteria demonstrated significant differences in OS between responders, CMR/PMR (56% ± 12%), compared to SMD (38% ± 17%, p=0.03) and PMD (21% ± 10%, p=0.01). Patients with progressive disease by both PERICST 1.0 and PSA response criteria demonstrated significantly worse OS (24-month OS: 0%, 12-month OS: 31% ± 14%) compared to patients with progressive disease by either response criteria.

**Conclusion**: PERCIST 1.0 may provide significant prognostic information for patients with mCRPC undergoing systemic chemotherapy, particularly when incorporated with PSA treatment response criteria.

## Introduction

Prostate cancer is the second leading cause of cancer-related death in men, affecting approximately 1 in 6 men. With the utilization of prostate-specific antigen (PSA) screening, the majority of patients diagnosed with prostate cancer present with locoregional disease 1. However, approximately 6% of patients present with metastatic disease on initial diagnosis and many patients with localized disease will ultimately develop recurrent and metastatic disease 2. The majority of patients with metastatic prostate cancer will eventually develop castrate-resistance, with progressive disease despite castrate serum androgen levels 3. Metastatic castrate-resistant prostate cancer (mCRPC) remains incurable and is associated with significantly shorter overall survival 4.

The accurate assessment of treatment response in patients with mCRPC is crucial 5. Early identification of non-responders ensure patients receive optimal management and avoid costly ineffective therapies, many of which have significant side effects 6. However, conventional methods for assessing treatment response, such as the Response Evaluation Criteria in Solid Tumors (RECIST) have limited value in mCRPC. The evaluation of osseous metastases is limited on conventional CT and the confounding flare phenomenon following treatment limits the utility of conventional bone scintigraphy 7.

Positron emission tomography (PET) has been gaining increasing traction in the imaging evaluation of prostate cancer. Several PET radiotracers, including ^18^F NaF, ^18^F- or ^11^C-choline, ^18^F-fluciclovine and prostate specific membrane antigen (PMSA)-based agents, have shown promising results in various phases of the disease 8-11. ^18^F-fluorodeoxyglucose (FDG), the most commonly utilized PET radiotracer for oncologic imaging, has shown mixed results for imaging patients with prostate cancer, with several studies showing low tumoral FDG uptake 12-14. However, many of these studies included cohorts of patients in the early stages of prostate cancer and may not be applicable to patients with more advanced metastatic disease. Indeed, several recent studies have demonstrated the utility of FDG in assessing patients with metastatic prostate cancer 15-18. Additionally, FDG PET has the inherent advantage of widespread availability and established use in treatment response criteria with the PET Response Criteria in Solid Tumors (PERCIST) 19.

The purpose of this single-center prospective cohort study was to evaluate the comparative prognostic utility of PERCIST 1.0 assessment using FDG PET/CT compared to conventional anatomy-based RECIST 1.1 and non-imaging PSA-based treatment response assessments in patients with mCRPC.

## Methods

### Patient Selection

Institutional Review Board and Radiation Safety Committee approvals were obtained for this prospective cohort study. All patients signed a written informed consent and the protocol was compliant with the Health Insurance Portability and Accountability Act. The investigation was performed under clinical trial registration number NCT00282906, “FDG Positron Emission Tomography and Computed Tomography (PET-CT) in Metastatic Prostate Cancer”.

Patients were prospectively recruited from 2005 to 2011. Patients with mCRPC were eligible for enrollment if they were beginning systemic medical therapy or transitioning to new systemic therapy after not responding to a prior treatment. Medical therapy, and the determination of castrate-resistant status, were made at the discretion of the treating physicians prior to enrollment into the study.

All patients underwent a baseline FDG PET/CT prior to the initiation of treatment and had a 4-month follow-up PET/CT after the start of therapy. Exclusion criteria included a history of malignancy other than prostate cancer, poorly controlled diabetes mellitus, active inflammatory conditions, active infections, and patients with recent or complicated nonhealing fractures or recent arthroplasty to diminish potential false positives. Patients with changes in baseline therapy greater than one week prior to the follow-up 4^th^ month PET/CT scan were also excluded.

### PET/CT Imaging

All patients underwent PET/CT imaging (Biograph Duo LSO; Siemens) 1 hour after intravenous administration of 370-550 MBq (10-15 mCi) of FDG, as previously described 16, 17. Customary quality control procedures were performed before all PET/CT scans (^68^Ge normalization daily and Society of Nuclear Medicine and Molecular Imaging PET/CT chest phantom every 3 months). All patients fasted for 4-6 h before ^18^F-FDG PET/CT imaging and water intake was encouraged before and after each scan. Blood glucose levels were obtained for all patients before intravenous administration of FDG and in all cases was less than 200 mg/dL.

Helical CT (pitch, 1.0; 90-130 mAs; 130 kVp) was performed first for each scan. Only oral contrast material was used. PET was then performed for 4 min per bed position at a sufficient number of bed positions to cover the top of the head to the feet. Raw CT data were reconstructed into 5 mm thick transverse images, and coronal and sagittal reformats were generated. CT-based attenuation-corrected PET images were reconstructed and viewed on a color high-resolution monitor. PET and CT images could be viewed on a continuous fusion scale from PET-only to CT-only images using E-soft image fusion software (Siemens).

PET/CT images were interpreted in consensus by two fellowship-trained board-certified nuclear radiologists with more than 20 years of experience in interpreting PET/CT studies. Lesions with visually discernible uptake and associated distinct correlation on CT, that were not physiologic or benign entities, were selected for further evaluation, with up to an arbitrary maximum of 30 lesions per scan for the various metastatic sites (e.g. bone, lymph node, soft tissue). The mean hepatic background standardized uptake value (SUV) was obtained for each patient by placing a 3-cm diameter ROI over an area of normal liver 20. The maximum SUV (SUVmax) of each lesion was than determined using 3-dimensional regions of interest (ROI) with vendor-provided software (Siemens) and corrected for lean body mass (SUL) 21. Lesions with a SULmax less than the average liver SUL were assigned a value of 0.

### Statistical Methods

Patients' response status at 4^th^ month compared to baseline was evaluated with three response criteria: RECIST 1.1, PERCIST 1.0 and PSA response criteria 2, 19, 22 (**Table 1**). For RECIST 1.1, up to 5 target lesions, with a maximum of 2 lesions per organ were identified with a short axis diameter of ≥15 mm for lymph nodes and a long axis diameter of ≥10mm for all other lesions as per RECIST 1.1 criteria 23. For patients without target lesions by RECIST 1.1, patients were categorized as CR if there was disappearance of all non-target lesions, non-CR/non-PD if there was persistence of one or more non-target lesions, and categorized as PD if there was any new lesion on follow-up examination. When available, bone scan data was used in conjunction with CT results for the detection of new osseous metastases and to identify resolution of previously active disease in sclerotic bone lesions. For PERCIST 1.0, up to 5 target lesions, lesions with a SULmax of at least 1.5 times greater than the liver SUL mean, with a maximum of 2 lesions/organ were identified. Non-target lesions with an SULmax between the liver SUL mean and 1.5 times the liver SUL mean were also evaluated. The PSA response criteria were based on those used in the Prostate Cancer Clinical Trials Working Group 3 (PCWG3) 24.

The associations between patients' response status and OS were evaluated. OS was defined as the duration in time between the dates of baseline scan and death. Patients who were alive at their last follow-up were censored at that time. Patients with different response status (CR/PR, SD, and PD) were compared with logrank tests. Due to a low number of patients with complete response and the primary objective of assessing responders (CR/PR) from non-responders, patients with complete and partial responses were grouped together for analysis. For RECIST 1.1 analysis, patients without target lesions categorized as non-CR/non-PD were grouped with the SD patients. Survival probabilities were calculated using the Kaplan-Meier method. Statistical analyses were performed using STATA software (version 11.0; StataCorp LP College Station, TX). All reported p-values were two-sided and a p value ≤0.05 was considered statistically significant.

## Results

**Figure 1:**
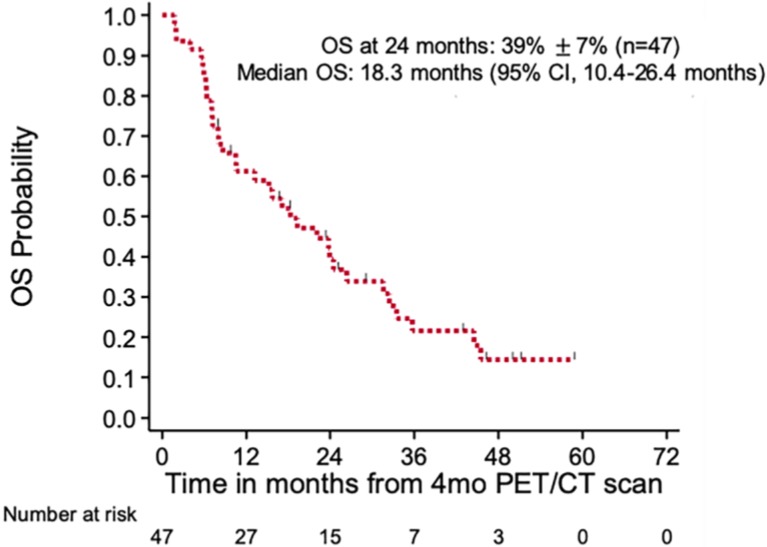
** Kaplan-Meier plot of overall survival of patients with metastatic castrate resistant prostate cancer.** CI- confidence interval, OS- overall survival.

A total of 87 patients with mCRPC were initially enrolled in this study, of which 53 received a 4^th^ month PET/CT. Of this cohort, 6 patients changed chemotherapy regimens more than a week before the 4^th^ month scan, leaving 47 patients eligible for analyses, two of which had a change of chemotherapy within the week of the PET/CT scan. **Table 2** summarizes patient and disease characteristics of the patient cohort. Of the 47 patients with mCRPC, 30 patients (63.8%) were chemotherapy naïve and 17 patients (36.2%) had been transitioned to a new chemotherapy regimen. The median OS of patients was 18.3 months (95% confidence interval [CI], 10.1-26.4 months), with the 24-month survival probability of 39% ± 7% (**Figure 1**).

**Figure 2:**
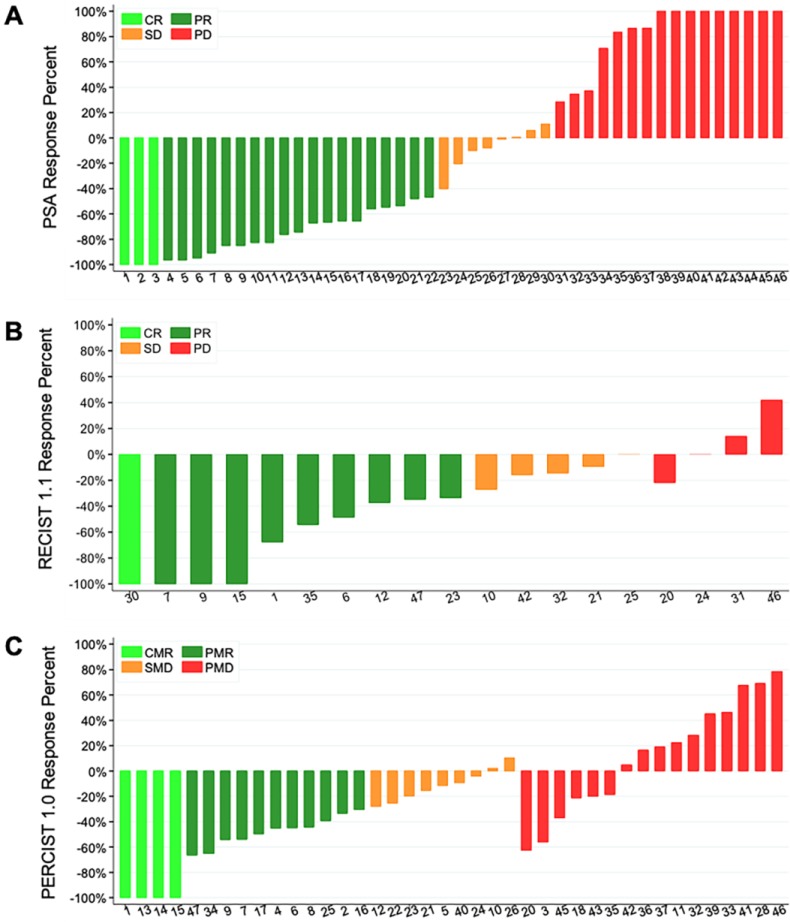
** Waterfall plots of the 4-month target lesions' percent of response after chemotherapy with PSA (A), RECIST 1.1 (B), and PERCIST 1.0 (C) response criteria.** Of note, only 19 of the 47 patients enrolled had target lesions by RECIST 1.1 criteria and were evaluable. CR/CMR- complete response, SD/SMD- stable disease, PD/PMD- progressive disease, PR/PMR- partial response.

**Figure 3:**
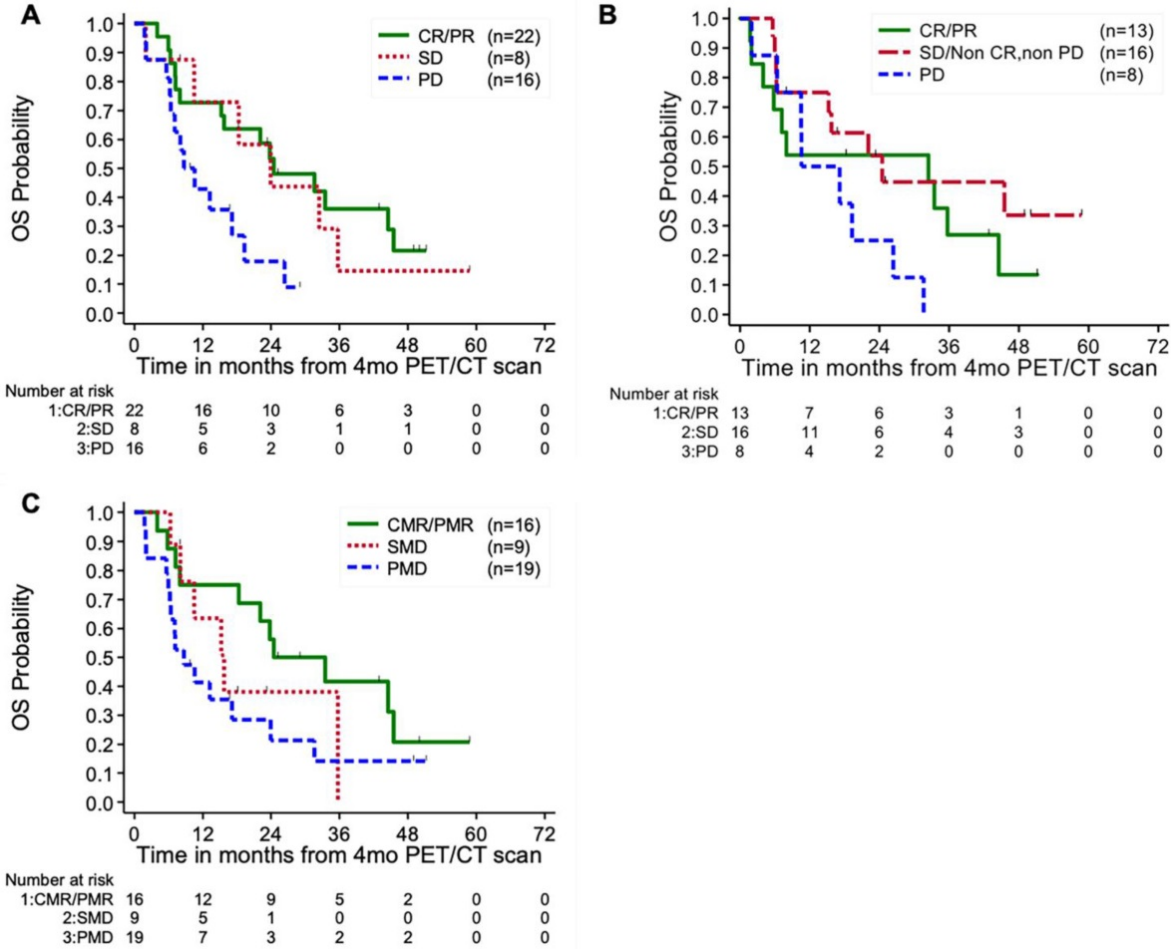
** Kaplan-Meier plot of overall survival by PSA (A), RECIST 1.1 (B), and PERCIST 1.0 (C) response status.** CR/CMR- complete response, SD/SMD- stable disease, PD/PMD- progressive disease, PR/PMR- partial response.

**Table 3** and **Figure 2** summarizes the results of the PSA, RECIST 1.1, and PERCIST 1.0 response criteria at the 4^th^ month imaging evaluation. Patients with progressive disease by PSA response criteria at 4 months demonstrated significantly worse OS (24-month survival probability: 18% ± 11%) compared to patients with stable disease (44% ± 19%, p=0.03) and complete or partial response (53% ± 11%, p=0.03) (**Figure 3a**). However, no significant differences were noted between the CR/PR and SD groups for this response criteria (p=0.83). RECIST 1.1 response criteria demonstrated a similar trend in OS, however no statistically significant differences were noted between patients with PD (25% ± 15%) compared to SD/non-CR, non-PD (54% ± 13%) and CR/PR (54% ± 14%) (p=0.13) (**Figure 3b**). PERCIST 1.0 criteria demonstrated significant differences in OS between responders, CMR/PMR (56% ± 12%), compared to SMD (38% ± 17%, p=0.03) and PMD (21% ± 10%, p=0.01), however no significant differences were noted between the PMD and SMD groups for this response criteria (p=0.79) (**Figure 3c**). No significant differences in OS were noted between patients with progressive disease by PSA response criteria compared to PERICST response criteria.

**Figure 4:**
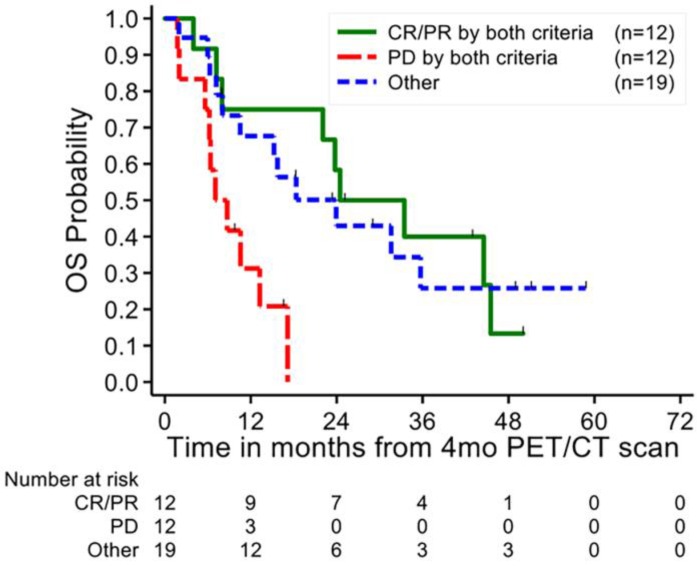
** Kaplan-Meier plot of overall survival of combined PERCIST 1.0 and PSA response status.** Patients with progressive disease by both response criteria demonstrated significantly worse overall survival compared to those categorized as progressive disease by one response criteria alone. CR/CMR- complete response, SD/SMD- stable disease, PD/PMD- progressive disease, PR/PMR- partial response.

A total of 12 patients (26%) demonstrated progressive disease by both PERCIST 1.0 and PSA based response criteria and 12 patients (26%) showed complete or partial response by both criteria (**Figure 4**). Patients with PD by both criteria demonstrated a significantly worse prognosis compared to PD by either response criteria individually, with a 24-month OS probability of 0% (p<0.01) and a 12-month OS probability of 31% ± 14% compared to 12-month OS probabilities of 43% ± 13% and 41% ± 11% (p<0.05) for PSA and PERCIST 1.0 criteria respectively. There were no significant differences between the partial and complete responders by both criteria (24-month OS 58% ± 14%) compared to the OS of responders by the individual criteria. Patients with mixed responses by PSA and PERCIST demonstrated a 24-month OS probability of 43% ± 12%, without a discernable trend in survival between discordant patients.

The RECIST 1.1 and PERCIST 1.0 response-criteria demonstrated concordances of 44% and 65%, respectively, with the PSA response criteria. RECIST 1.1 was concordant with PERCIST 1.0 criteria for 50% of cases. The main discrepancies between RECIST 1.1 criteria compared to the PSA and PERCIST 1.0 criteria occurred due to the inability of RECIST 1.1 to assess differences in complete and partial responses from those with stable or non-progressive/non-partial responsive disease. 38 patients (62%) demonstrated complete or partial response in the PSA group and 28 patients (46%) in the PERCIST 1.0 group compared to only 13 patients in the RECIST 1.1 group (35% of evaluable patients).

Of the 47 patients enrolled in this study, 30 were chemotherapy naïve and 17 were enrolled after switching from a prior chemotherapy agent. The chemotherapy naïve patients demonstrated better overall survival (58% ± 9%) compared to patients enrolled switched to a new treatment (7% ± 7%, p=0<0.001). Chemotherapy naïve patients demonstrated similar trends in OS compared to the overall cohort, potentiually due to low sample size.

## Discussion

PSA-based response criteria play an important role treatment monitoring for patients with mCRPC. Studies have shown shorter PSA doubling times and high PSA velocity are associated with significantly shorter OS in patients with mCRPC 25, 26. However, there are several limitations in using PSA data for patients with mCRPC 27. Docetaxel, a common first line chemotherapy agent for mCRPC patients, has been shown to down regulate PSA expression and secretion, resulting in discrepancies between PSA changes and disease response 28. In addition, up to 20% of patients on docetaxel therapy have been shown to have a PSA flare during the initiation of treatment, despite ensuing therapeutic response 29. Furthermore, mCRPC can demonstrate significant intra- and inter-tumor heterogeneity with varying degrees of PSA expression and chemosensitivity, thus complicating the interpretation of PSA data on overall treatment impact in patients with multiple sites of disease 30. Lastly, with the increasing use of molecularly targeted and other novel agents, (e.g. ^223^Ra dichloride, sorafenib, cabozantinib, sipuleucel-T), there may be dissociated anti-tumor and PSA effects, and therefore, the utility of PSA-based response assessment will be limited 31, 32.

PERCIST 1.0, introduced by Wahl et al. in 2009 as a guideline to assess treatment response in patients with cancer using FDG PET, has since been evaluated for the use in numerous malignancies, including colorectal cancer, small-cell lung cancer, and lymphoma 33. Although no studies to date have evaluated the utility of PERCIST 1.0 for patients with mCRPC, there are a few studies which have investigated the use of a modified PERCIST criteria utilizing ^68^Ga-PMSA-11 PET/CT. In a study of 88 patients with biochemically progressive metastatic prostate cancer, Gupta et al. investigated the utility of a modified PERCIST using changes in uptake value on ^68^Ga-PMSA-11 PET/CT compared to RECIST 1.1. In this study, the modified PERCIST classified a significantly higher number of patients with disease progression compared to RECIST 1.1 (80.7% PD by PERCIST vs 50.6% PD by RECIST 1.1, p<0.05) 34. Seitz et. al demonstrated similar results with a modified PERCIST criteria using ^68^Ga-PMSA-11 PET/ CT showing high concordance between PERCIST and PSA-based response compared to RECIST 1.1 35.

In our study we found that PERCIST 1.0 treatment response assessment at 4 months correlated highly with PSA response criteria and had significant implications for predicting OS. Of the response criteria, PERCIST 1.0 was superior at distinguishing the overall survival of responders compared to patients with stable disease, whereas the PSA response criteria was superior at distinguishing the overall survival of patients with progressive versus stable disease. However, the PSA response criteria lacked the ability to distinguish survival differences in patients with stable vs. responsive disease. RECIST 1.1, which incorporates the use of CT data, showed a trend in distinguishing the OS of progressors vs. non-progressors, although not statistically significant in our study, yet was unable to distinguish survival differences between patients with responsive versus stable disease. This may be in large part due to the inherent inability of RECIST 1.1 to accurately monitor osseous metastases, which constitutes the majority of lesions in patients with mCRPC. When lacking target lesions, RECISTS 1.1 cannot distinguish patients with stable disease from those with partial response. These findings highlight the added value that FDG PET contributes to conventional imaging with regards to assessing treatment response and disease aggressiveness. This added information may be valuable to treating clinicians, potentially prompting earlier changes in systemic therapies in nonresponding patients, which may lead to improved overall outcome. Of note, patients with progressive disease by both PERCIST 1.0 and PSA response criteria demonstrated significantly worse OS compared to those with progressive disease by one criterion alone. These combined results may be useful in identifying patients with highly aggressive disease, providing crucial prognostic information to clinicians and potentially prompting the use of more aggressive treatment regimens.

Potential limitations for this study include grouped analysis of patients undergoing first-line chemotherapy vs those enrolled during a transition to a new agent. However, the majority of the patients enrolled in this study underwent first-line chemotherapy and subset analyses of the chemotherapy naive patients demonstrated similar trends in OS. As may have been expected, the cohort of patients switched to new chemotherapy agents demonstrated significantly worse OS, reflecting more aggressive tumor biology 36. While none of the response criteria were able to demonstrate significant differences in OS between response groups in this cohort, this may be secondary to a low sample size and additional larger studies are required to evaluate the effectiveness of the response criteria in this subset of patients. In addition, although we used RECIST 1.1 response criteria in conjunction with available bone scan data, follow-up bone scans were not performed in all patients to exclude lesions secondary to the flare phenomenon. However, only 4 patients were classified as PD by RECIST 1.1 criteria due to isolated bone scan findings, 3 of which demonstrated corresponding lesions on FDG PET/CT. To accurately assess the treatment response between the baseline and 4-month follow-up, only patients with no change in chemotherapy regimens between baseline and follow-up scans were included, excluding the assessment of patients who underwent early changes in therapy at the discretion of their treating physician. Lastly, due to practical and ethical constraints there was a lack of histologic verification for the metastatic lesions. However, all selected lesions for analysis were required to have correlation on CT to be considered as sites of metastatic disease.

## Conclusion

PERCIST 1.0 may provide useful prognostic information in men with mCRPC undergoing treatment, particularly at distinguishing the overall survival in patients with favorable treatment response compared to those with stable disease. Furthermore, the incorporation of both PERCIST 1.0 and PSA-based response criteria may be useful for identifying patients with highly aggressive disease and poor overall survival, although further studies are required to confirm these results.

## Figures and Tables

**Table 1:** Treatment Response Criteria

PSA-based response criteria		RECIST 1.1	PERCIST 1.0
CR/CMR	PSA decline to undetectable (<0.2 ng/mL)	Disappearance of all target and non-target lesions	Disappearance of all FDG avid lesions
PR/PMR	≥50% PSA decline and ≥2 ng/mL decline	≥30% decrease in sum of target lesions	≥30% decrease in SUL peak + decline by ≥0.8 SUL
SD/SMD	Neither PR or PD	Neither PR or PD	Neither PR or PD
PD/PMD	≥25% PSA increase and ≥2 ng/mL increase	≥20% increase in sum of target lesions + absolute increase of at least 5 mm or new lesions	≥30% increase in SUL peak + >0.8 SUL increase or new lesions

**^1^** CR=complete response, PR=partial response, SD=stable disease, PD=progressive disease. **^1^** CMR=complete metabolic response, PMR=partial metabolic response, SMD=stable metabolic disease, PMD=progressive metabolic disease. ^2^ SUL= Standard uptake value, corrected using lean body mass

**Table 2:** Patient and Disease Characteristics

Variables	Metastatic castrate-resistant prostate cancer (N=47)	
	n or median	%
**Age at PET/CT Scan (years)**		
Median (Min, 25%, 75%, Max)	69.(50, 60, 74, 89)	
50-69	24	51.1
70-90	23	48.9
**Race/Ethnicity**		
Hispanic	10	21.3
White	32	68.1
Other	5	10.6
**Years since initial diagnosis (years)**		
Median (Min, 25%, 75%, Max)	7.6 (0.47, 2.8, 11.8, 17.6)	
**Gleason Score at diagnosis**		
Median (Min, 25%, 75%, Max)	8 (5, 7, 9, 10)	
Missing (number of patients)	5	
**Prior Chemotherapy**		
No	30	63.8
Yes	17	36.2
**PSA (ng/ml) at baseline**		
Median (Min, 25%, 75%, Max)	46.4 (0.09, 16.5, 99.8, 4530)	
**Alkaline Phosphatase**		
Median (Min, 25%, 75%, Max)	94 (36, 65, 148, 847)	
**Sites of Disease**		
Bone Only	20	42.6
Lymph Nodes Only	6	12.7
Soft Tissue Only	1	2.1
Lymph Nodes and Soft Tissue Only	3	6.4
Bone and Lymph Nodes Only	11	23.4
Bone and Soft Tissue Only	3	6.4
Bone, Lymph Nodes and Soft Tissue	3	6.4

**Table 3:** PCWG2-PSA, RECIST 1.1, PERCIST 1.0 response evaluation at 4^th^ month follow-up

Variables		Metastatic castrate-resistant prostate cancer (N=47)	
		n or median	%
**4^th^ Month Disease Evaluation: PSA**			
	CR	3	6
	PR	19	40
	SD	8	17
	PD	16	34
	PSA <0.2 at Baseline and 4^th^ month	1	2
**4^th^ Month Disease Evaluation: RECIST 1.1 ^1^**			
** # of Patients WITH Target Lesions at Baseline**		19	51
# of Target Lesions: Median (Min, 25%, 75%, Max)		2 (1, 1, 3, 5)	
# of Non-Target Lesions: Median (Min, 25%, 75%, Max)		5 (0, 2, 10, 21)	
Total # of Lesions: Median (Min, 25%, 75%, Max)		8 (1, 4, 11, 22)	
Overall Response Evaluation			
	CR	1	
	PR	9	
	SD	5	
	PD	4	
** # of Patients WITHOUT Target Lesions**		18	49
# of Non-Target Lesions: Median (Min, 25%, 75%, Max)		5 (0, 1, 10, 20)	
Response Evaluation (non-Target lesions)			
	CR	3	
	Non-CR/non-PD	11	
	PD	4	
**4^th^ Month Disease Evaluation: PERCIST 1.0 ^1^**			
# of Target Lesions: Median (Min, 25%, 75%, Max)		3 (0, 1, 5, 5)	
# of Non-Target Lesions: Median (Min, 25%, 75%, Max)		3 (0, 0, 6, 18)	
Total # of Lesions: Median (Min, 25%, 75%, Max)		6 (0, 2, 11, 23)	
MAX SUL: Median (Min, 25%, 75%, Max)		3.6 (0, 2.1, 5.1, 10.6)	
Overall Response Evaluation			
	CMR	4	9
	PMR	11	23
	SMD	10	21
	PMD	19	40
	Not Evaluable by PERCIST 1.0 ^2^	3	6
	Occurrence of New Lesions	12	26

^1^ For patients with extensive number of bone lesions, not all bone lesions were counted or measured for disease evaluation. ^2^ Patients not evaluable with PERCIST 1.0 due to not having any target or non-target lesions or new lesions based on PERCIST 1.0.

## Abbreviations

CT: computed tomography
PET: positron emission tomography
FDG: ^18^F-fluorodeoxyglucose
PSA: prostate-specific antigen
PSMA: prostate specific membrane antigen
CI: confidence interval
HR: hazard ratio
SE: standard error
mCRPC: metastatic castrate-resistant prostate cancer
CR: complete response
PR: partial response
SD: stable disease
PD: progressive disease
CMR: complete metabolic response
PMR: partial metabolic response
SMD: stable metabolic disease
PMD: progressive metabolic disease
RECIST: Response Evaluation Criteria in Solid Tumors
PERCIST: PET Response Criteria in Solid Tumors
SUL: standardized uptake value corrected for lean body mass
